# Association of Occupational and Leisure-Time Physical Activity with Aerobic Capacity in a Working Population

**DOI:** 10.1371/journal.pone.0168683

**Published:** 2017-01-03

**Authors:** Jonas Mundwiler, Ulla Schüpbach, Thomas Dieterle, Jörg Daniel Leuppi, Arno Schmidt-Trucksäss, David Paul Wolfer, David Miedinger, Stefanie Brighenti-Zogg

**Affiliations:** 1 University Clinic of Internal Medicine, Cantonal Hospital Baselland, Liestal, Switzerland; 2 Department of Sport Exercise and Health, University of Basel, Basel, Switzerland; 3 Faculty of Medicine, University of Basel, Basel, Switzerland; 4 Department of Health Sciences and Technology, ETH Zurich, Zurich, Switzerland; 5 Institute of Anatomy, University of Zurich, Zurich, Switzerland; 6 Department of Occupational Medicine, Swiss National Accident Insurance Fund (Suva), Lucerne, Switzerland; University of France Comté, FRANCE

## Abstract

**Introduction:**

Objective data on the association of maximal aerobic capacity (VO_2max_) with work related physical activity are sparse. Thus, it is not clear whether occupational physical activity (OPA) contributes to an increase of VO_2max_. This study examined the association of VO_2max_ with work and non-work related physical activity in a Swiss working population.

**Methods:**

In this cross-sectional study, a total of 337 healthy and full-time employed adults were recruited. Demographic data, height, weight and BMI were recorded in all subjects. Participants were classified into nine occupational categories (ISCO-88) and merged into three groups with low, moderate, and high OPA. Physical activity was objectively measured by the SenseWear Mini Armband on seven consecutive days (23 hours per day). Participants were regarded as sufficiently active when accumulating ≥30 min of moderate-to-vigorous physical activity per day. VO_2max_ was evaluated using the multistage 20-meter shuttle run test.

**Results:**

Data of 303 participants were considered for analysis (63% male, age 33 yrs, SD 12). Multiple linear regression analysis (adjusted R^2^ = 0.69) revealed significant positive associations of VO_2max_ with leisure-time physical activity (LTPA) at vigorous intensity (β = 0.212) and sufficient moderate-to-vigorous physical activity (β = 0.100) on workdays. Female gender (β = -0.622), age (β = -0.264), BMI (β = -0.220), the ratio of maximum to resting heart rate (β = 0.192), occupational group (low vs. high OPA, β = -0.141), and smoking (β = -0.133) were also identified as independent predictors of VO_2max_.

**Conclusions:**

The present results suggest that VO_2max_ is positively associated with LTPA, but not with OPA on workdays. This finding emphasizes the need for employees to engage in sufficient high-intensity physical activity in recreation for maintaining or improving VO_2max_ with regard to health benefits.

## Introduction

VO_2max_ is defined as the maximum rate of oxygen consumption. It is generally accepted as an appropriate measure of the functional capacity of the cardiorespiratory system and is commonly interpreted as an index of cardiorespiratory fitness [1]. Previous studies found that poor cardiorespiratory fitness was a risk factor for various diseases such as hypertension, stroke, type 2 diabetes, and metabolic syndrome [2, 3]. Other studies reported that a low VO_2max_ was associated with all-cause mortality and mortality from cardiovascular disease [4].

VO_2max_ is determined by genetic factors, age, gender, as well as physical activity, body fat, smoking, and medical conditions, i.e. metabolic syndrome and diabetes [5]. VO_2max_ decreases with age with an average rate of decline of about 1% per year or 10% per decade after the age of 25 [6, 7]. Recent data suggest that the ability of an individual to increase VO_2max_ is genetically determined. Each individual disposes of a predetermined genetic window, and can vary the amount of VO_2max_ with exercise training or detraining within that window [8]. VO_2max_ values range from about 10 ml/kg/min in severely ill cardiac patients to 80–90 ml/kg/min in world-class runners and cross-country skiers [9]. VO_2max_ may be substantially increased in response to endurance training [8].

For decades, governmental and non-governmental agencies have promoted physical activity for individuals’ health benefits. The World Health Organization recommends that adults between 18 and 64 years should engage in ≥30 min of at least moderate-intensity physical activity on most days of the week. Aerobic activity should be performed in bouts of ≥10 min duration across different domains such as work, leisure-time, transport, domestic and garden [10]. Since most people in full-time employment spend one third or more of the day at work, it is conceivable that occupational physical activity (OPA) may contribute to a large extent to total daily activity. While leisure-time physical activity (LTPA) is well known to be positively associated with VO_2max_ [8], only few data are available on the relationship between OPA and VO_2max_ in employees. With regards to LTPA, Ong & Sothy [11] found that regularly exercising men in sedentary occupations had a significantly higher mean VO_2max_ than non-regularly exercising counterparts. In contrast, the potential positive effects of OPA on VO_2max_ are less well investigated [12]. However, available data suggest that OPA, independent of formal exercise programs, may positively affect VO_2max_ [13].

Previous studies measuring physical activity in employees used pedometers and accelerometers in combination with self-reported questionnaires. Self-reported questionnaires are the most used method for physical activity assessment [14]. However, validation studies comparing self-reported estimates to the gold standard method Doubly-Labeled-Water (DLW) are inconsistent [15, 16]. Due to accuracy, ability to capture large amounts of data and ease of administration, accelerometers are widely used today [17]. However, they are not accurate in estimating physical activity during activities involving uphill and downhill walking or when carrying heavy load while walking [18]. Pedometers directly observe duration of activities, but do not record intensity and frequency of physical activity or purely upper body movements [19]. Armband devices, such as the SenseWear Mini Armband, integrate motion and heat-related sensors. This dual measurement strategy is more sensitive for assessing energy expenditure associated with complex and non-ambulatory activities, such as carrying heavy load while walking [19]. Furthermore, this method ensures a sensitive determination of acceleration provoked by muscle power or externally by a vehicle or gravitation [20].

Due to methodological limitations and lack of studies, potential associations of objective physical activity and VO_2max_ in employees still need to be clarified by further investigations. Furthermore, with regards to the development of evidence-based activity recommendations it is important to know, whether OPA contributes to an improvement of VO_2max_. Therefore, the objective of this study was to analyse the relationship between VO_2max_ and work and non-work related physical activity as measured by the SenseWear Mini Armband in a Swiss working population. The secondary objective was to evaluate the effect of demographic factors on VO_2max_ including gender, age, body mass index (BMI), and smoking.

## Materials and Methods

### Study Participants

From May 17, 2013 (first participant in) to February 11, 2015 (last participant in), a total of 337 healthy and at minimum 80% employed adult workers from various companies of the Basel region, Switzerland were recruited. Exclusion criteria were insufficient knowledge of the German language, movement restrictions as well as diseases and accidents within the last three months that affected productivity at the workplace. Furthermore, individuals, who had to comply with specific security regulations, and night shift workers could not take part in this study because of their altered sleep, eating and physical activity behaviour. This investigation has been conducted according to the Declaration of Helsinki and was approved by the local ethics committee “Ethikkommission Nordwest- und Zentralschweiz” (EKNZ, 260/12) on December 21, 2012. Written informed consent was obtained from all study participants prior to study entry.

### Study Design and Procedures

In this cross-sectional study, the aim was to recruit an equal distribution of subjects across different occupational groups. A permit from leading persons of miscellaneous companies was requested to receive contact details for potentially recruitable employees, who were then informed and asked for study participation by phone or by e-mail. The selected companies included medium sized corporations from the public sector (e. g. hospitals) as well as small sized private firms (e. g. construction companies). At the study visit, height and weight were reliably measured. Height was assessed without shoes by a medical measuring stick to the nearest mm (model Seca 217, measurement range: 20 to 205 cm, Seca AG, Reinach, Switzerland). The measurement of weight was performed on subjects in light clothing without shoes by a medical scale with an accuracy of 0.1 kg (model Seca 877, load capacity: 200 kg, Seca AG, Reinach, Switzerland). BMI was calculated from measured height and weight (BMI = weight/height^2^ [kg/m^2^]). Subjects with a BMI of 25 kg/m^2^ or more were classified as overweight, and those with a BMI of 30 kg/m^2^ or more as obese [21]. In addition, a variety of personal and job-related factors were recorded by a generic questionnaire, such as age, gender, nationality, marital status, smoking status, alcohol consumption, highest education, current profession, daily working hours, working time model, medication, psychotherapy, illnesses and accidents within the last three months. The reported professions were classified into nine categories based on the International Standard Classification of Occupations 1988 (ISCO-88) [22]. Participants were then merged into three groups with low (managers, scientists, office workers), moderate (technicians, service workers, machine operators), and high (agricultural workers, craftsmen, labourers) OPA [23]. Prior to the observation period, subjects performed a 20-meter shuttle run test in order to determine VO_2max_. During the subsequent week, participants were instructed to wear the SenseWear Mini Armband on seven consecutive days in order to objectively measure daily physical activity.

### Measurements

#### 20-meter shuttle run test

The multistage 20-meter shuttle run test is a common endurance fitness test to evaluate the maximal aerobic capacity of healthy adults. It is simple in use, economical and large groups can be tested simultaneously. Validity of the one-minute stage version of the 20-meter shuttle run to predict VO_2max_ in adults was established by Léger & Gadoury [24], who compared the maximal shuttle run speed to VO_2max_ attained during a multistage treadmill test (r = 0.90). Test-retest reliability was found to be very high (r = 0.95) in healthy adults [23].

This test was conducted on a flat, non-slip surface. Participants were instructed to run back and forth between two lines, which were 20-meters apart, with a running velocity determined by audio signals [23]. Starting speed was 8.5 km/h and every minute (stage), speed was increased by 0.5 km/h until the subject could no longer keep the pace and did not reach the lines in time twice in a row [23]. The test result corresponded to the number of reached stages. According to a validated table [25], this score was used to predict VO_2max_, which could be compared to age-dependent normative data for males and females.

Resting heart rate (HR_rest_), systolic and diastolic blood pressure were assessed prior to testing after 10 minutes at rest in a sitting position in a quiet environment. Heart rate was continuously recorded during the test up to maximum frequency (HR_max_) and recovery pulse (HR_recovery_), systolic and diastolic blood pressure were measured two minutes after the end of the test (in a sitting position in a quiet environment). The ratios of HR_max_-to-HR_rest_ and HR_max_-to-HR_recovery_ were calculated as (HR_max_/HR_rest_) and (HR_max_/HR_recovery_) [26], respectively. Hypertension was defined as a blood pressure of ≥140/90 mmHg. Four participants did not perform the 20-meter shuttle run due to a resting systolic blood pressure >180 mmHg. They were pairwise excluded from the corresponding analyses.

#### SenseWear mini armband

The SenseWear Mini Armband (Model MF-SW) is a small, lightweight and wireless multisensory activity monitor developed by BodyMedia Inc., Pittsburgh, Pennsylvania, USA (now Jawbone Inc., San Francisco, California, USA), which integrates motion data from a three-axis accelerometer along with other sensors such as heat flux, skin temperature and galvanic skin response. Validity of the SenseWear Mini Armband was established by Johannsen *et al*. [20] comparing energy expenditure estimates of the SenseWear Mini Armband against the criterion method DLW in healthy adults. The Armband showed a high intraclass correlation with DLW (r = 0.85) and a low absolute error rate (8%, SD 7%) [20].

Subjects were instructed to wear the SenseWear Mini Armband on the upper left arm (triceps area) for seven consecutive days, including while sleeping, with the exception of the time spent on personal hygiene. The first and the last incomplete measurement day, including the study visits, were not taken into account. Therefore, the investigated measurement period was five days, which had to consist of at least three workdays to be included in the analysis [27]. A day was considered as a whole workday, if participants worked cumulatively ≥6 h, and as a half workday in case of ≥3 to <6 h. Days with <3 working h were regarded as non-working days. Measurement periods of <22 h per day or <12 h during wake time were excluded from analysis [28]. Information about workdays and non-working days as well as work-time and leisure-time on workdays was obtained from diaries participants filled in during the measurement period.

#### Calculation of physical activity scores

The physiological data, collected by the armband’s sensors, were processed by specific algorithms available in the SenseWear software (BodyMedia, professional software V.7.0, algorithm V.2.2.4). Patients’ average daily number of steps, active energy expenditure (AEE), physical activity level in metabolic equivalents of task (METs) and physical activity duration at different intensities were examined. One MET corresponds to 3.5 ml/min/kg VO_2_ [29]. For all variables, average values were calculated separately for workdays and non-working days as well as for work-time and leisure-time on workdays. The amount of physical activity (min/day) at a certain intensity level was calculated in two ways (Table 1). First, one-minute intervals in which the intensity reached the following MET thresholds were summed up: moderate physical activity (MPA) ≥3 to <6 METs, high physical activity (HPA) ≥6 to <9 METs, and very high physical activity (VHPA) ≥9 METs. As participants performed only little physical activity in the VHPA range, this variable has been combined with HPA for a more representative measure with respect to the regression analysis (combined variable: vigorous physical activity (VPA)). Second, because current guidelines suggest accumulating physical activity bouts of ≥10 min [29], this was considered in the calculation of moderate-to-vigorous physical activity (MVPA) ≥3 METs. Thus, we were able to investigate whether participants fulfilled the recommendation of MVPA of ≥30 min per day calculated from bouts ≥10 min on workdays and non-working days [30].

**Table 1:** List of SenseWear physical activity variables and their definition.

Variable	Definition
**MPA**	Sum of **m**oderate (one-minute intervals) **p**hysical **a**ctivity (≥3 to <6 METs) during work-time and during leisure-time on workdays / on non-working days.
**HPA**	Sum of **h**igh (one-minute intervals) **p**hysical **a**ctivity (≥6 to <9 METs) during work-time and during leisure-time on workdays / on non-working days.
**VHPA**	Sum of **v**ery **h**igh (one-minute intervals) **p**hysical **a**ctivity (≥9 METs) during work-time and during leisure-time on workdays / on non-working days.
**VPA**	Sum of **v**igorous (one-minute intervals) **p**hysical **a**ctivity (≥6 METs) (combination of HPA and VHPA, produced for the regression analysis).
**MVPA**	Sum of sufficient (≥30 min) / insufficient (<30 min) **m**oderate-to**-v**igorous (≥3 METs) **p**hysical **a**ctivity (bouts of ≥10 min) on workdays / non-working days.

### Statistical Analysis

Data were analyzed using IBM SPSS Statistics (version 22.0). Significance was set at the 5% level. The Shapiro-Wilk test was used to test whether data were normally distributed. Data are presented as mean and standard deviation (SD) or number and percentage. To analyse differences across gender and physical activity categories, mean comparisons were performed using Student’s T-test or Mann-Whitney test, if appropriate. Categorical data were analyzed with Chi-Square test. To identify potential predictors of VO_2max_, a multiple linear regression analysis was performed using the backward stepwise method. VO_2max_ was considered as dependent variable. Independent variables were age, gender, BMI, AEE / MPA / VPA / Steps during work-time, leisure-time and on non-working days, MVPA on workdays and non-working days (insufficient vs. sufficient), HR_max_-to-HR_rest_, smoking (never smokers vs. current smokers / never smokers vs. ex-smokers), and occupational group (group 1 vs. group 2 / group 1 vs. group 3). Validity of the regression model was established by checking essential assumptions. Checks for collinearity were made using tolerance and variance inflation factor (VIF) for each of the independent variables calculated by means of the collinearity diagnostics in IBM SPSS Statistics. AEE measured by the SenseWear Mini Armband was subject to power calculation. Assuming a sample size of 100 subjects in each occupational group, there is a power of >90% to detect a mean difference of 500 kcal between any of these groups. This calculation was based on the assumption of a within group SD of 730 kcal and on a two-sided significance level of 5% [31].

## Results

### Subjects’ Characteristics

Of the 337 recruited subjects 303 were considered for analysis. Descriptive data for total (n = 303), male (n = 190, 63%), and female (n = 113, 37%) subjects are presented in Table 2. 31% of subjects (n = 95) were found to be overweight and 7% (n = 21) were obese. Age ranged from 18 to 61 years and did not differ significantly between sexes. A higher percentage of males compared to females were current smokers, while more women than men were ex-smokers. BMI, HR_max_, HR_recovery_, and systolic blood pressure were significantly increased in male subjects. In contrast, only HR_rest_ was higher in females. Diastolic blood pressure was about the same in men and woman. More than half of the men suffered from hypertension, whereas in woman less than one-third was affected. Gender distribution was more or less balanced in group 1 (low OPA) and group 2 (moderate OPA), while only 4% of the investigated females were represented in group 3 (high OPA).

**Table 2:** Characteristics of study subjects.

Variables	Total (n = 303)	Male (n = 190)	Female (n = 113)
	Mean (SD) or N (%)	Mean (SD) or N (%)	Mean (SD) or N (%)
Age [yrs]	33 (12)	33 (13)	35 (12)
BMI [kg/m^2^]	24 (3)	25 (3) ***	23 (4)
Current smokers	64 (21%)	46 (24%)	18 (16%)
Ex-smokers	61 (20%)	32 (17%)	28 (25%)
HR_rest_	72 (13)	70 (13)*	74 (13)
HR_max_	183 (15)	187 (14) ***	178 (15)
HR_recovery_	106 (15)	108 (14) **	102 (16)
Sys BP_rest_ [mmHg]	135 (15)	140 (12)**	128 (18)
Dia BP_rest_ [mmHg]	82 (10)	82 (10)	80 (11)
Hypertension (≥140/90 mmHg)	130 (43%)	104 (55%)	26 (23%)
History of Diabetes	0 (0%)	0 (0%)	0 (0%)
History of CAD	1 (0%)	1 (0%)	0 (0%)
Group 1 (low OPA)	101 (33%)	55 (29%)	46 (41%)
Group 2 (moderate OPA)	102 (34%)	40 (21%)	62 (55%)
Group 3 (high OPA)	100 (33%)	95 (50%) **	5 (4%)

BMI, body mass index; CAD, coronary artery disease, Dia BP_rest_, diastolic blood pressure at rest; HR_max_, maximum heart rate; HR_recovery_, recovery pulse (measured two minutes after the end of the 20-meter shuttle run test); HR_rest_, heart rate at rest; OPA, occupational physical activity; SD, standard deviation; Sys BP_rest_, systolic blood pressure at rest; VO_2max_, maximal oxygen consumption during multistage 20-meter shuttle run test.

* p<0.05,

** p<0.01,

***p<0.001 (two-tailed) vs. females.

Thirty-four subjects (10%) have worn the SWMA on less than three workdays and were therefore excluded from the entire analysis. Reasons for non-wearing or non-evaluation were: technical problems (n = 4), illness during observation period (n = 2), no paid occupation (n = 5), no interest (n = 15), loss of the armband (n = 4), skin irritations (n = 2) or sleep problems (n = 2). Another 24 individuals had missing SWMA data on non-working days due to more workdays during observation period and were pairwise excluded from the corresponding analyses.

### Physical Activity Data

Table 3 illustrates VO_2max_ and objective physical activity measured by the SenseWear Mini Armband according to gender. Males had a significantly higher VO_2max_ than females. Overall, most physical activity was performed within the moderate-intensity range. Furthermore, activity levels at all intensities, AEE, and the number of daily steps were higher in males compared to females. Activity parameters differed significantly between sexes on workdays during work- and leisure-time (with the exception of VHPA during leisure-time), while no gender-dependent differences in physical activity were found on non-working days (with the exception of AEE). Moreover, men showed higher physical activity levels on workdays (work-time and leisure-time added) than on non-working days. In women, this was the case for MPA, AEE, and steps, whereas it was the contrary for HPA and VHPA.

**Table 3:** Physical activity data, measured with objective methodology, SenseWear Mini Armband.

Variables		Total (n = 303)	Male (n = 190)	Female (n = 113)
		Mean (SD)	Mean (SD)	Mean (SD)
VO_2max_	[ml/kg/min]	40 (10)	45 (8) ***	33 (7)
MPA	Work-time [min/day]	151 (131)	190 (143) ***	85 (70)
	Leisure-time [min/day]	107 (50)	113 (52) *	98 (44)
	Non-working day [min/day]	178 (105)	187 (110)	164 (94)
HPA	Work-time [min/day]	8 (14)	11 (16) ***	2 (6)
	Leisure-time [min/day]	9 (11)	10 (11) **	7 (11)
	Non-working day [min/day]	12 (18)	13 (21)	9 (13)
VHPA	Work-time [min/day]	0 (1)	0 (2) **	0 (0)
	Leisure-time [min/day]	2 (5)	2 (5)	1 (4)
	Non-working day [min/day]	2 (7)	2 (8)	2 (6)
AEE	Work-time [kcal/day]	795 (816)	1068 (905) ***	337 (273)
	Leisure-time [kcal/day]	609 (321)	700 (341) ***	456 (211)
	Non-working day [kcal/day]	950 (632)	1089 (706) ***	724 (398)
Daily steps	Work-time	6521 (3888)	7511 (4292) ***	4855 (2285)
	Leisure-time	5638 (2592)	5470 (2727) *	5919 (2332)
	Non-working day	8997 (6190)	9187 (7321)	8685 (3673)

AEE, active energy expenditure; MPA / HPA / VHPA, physical activity duration at moderate (3–6 METs) / high (6–9 METs) / very high (≥9 METs) intensity; SD, standard deviation; VO_2max_, maximal oxygen consumption during multistage 20-meter shuttle run test.

* p<0.05,

** p<0.01,

***p<0.001 (two-tailed) vs. females.

### VO_2max_ across Categories of Physical Activity

On workdays, VO_2max_ was significantly higher in participants, who were sufficiently active (≥30 min MVPA/day, 42 ml/min/kg, SD 9, n = 251, 84%) compared to those, who were insufficiently active (<30 min MVPA/day, 31 ml/min/kg, SD 7, n = 48, 16%) (p<0.01). However, on non-working days, the difference between sufficiently (41 ml/min/kg, SD 9, n = 208, 75%) and insufficiently (37 ml/min/kg, SD 10, n = 71, 25%) active participants was not significant (p = 0.129).

Regarding the combination of physical activity categories on workdays and non-working days, VO_2max_ was found to be lowest in the category, which did not fulfil the activity recommendations on both, workdays and non-working days (30 ml/min/kg), slightly increased in the category, which fulfilled the recommendations on non-working days, but not on workdays (34 ml/min/kg), clearly higher in those, who were sufficiently active on workdays, but not on non-working days (41 ml/min/kg) and highest in the category, which fulfilled the recommendations on both, workdays and non-working days (42 ml/min/kg).

### VO_2max_ Reference Values

Average VO_2max_ values stratified by age and gender are given in Table 4. They were found to be well within the representative reference ranges of VO_2max_ for non-athletes provided by Kenney *et al*. [8].

**Table 4:** VO_2max_ reference values according to age and gender.

		VO_2max_ [ml/kg/min]	Decrease over decade	VO_2max_ [ml/kg/min] ranges for non-athletes according to Kenney *et al*. [8]
Age categories	N (%)	Mean (SD)	%	Mean (reference ranges)
**Total:**	**303**			
18–29 yrs	131 (43)	43 (9)		
30–39 yrs	77 (26)	41 (9)	- 5%	
40–49 yrs	52 (17)	39 (8)	- 5%	
50–61 yrs	43 (14)	33 (8)	-15%	
**Male:**	**190**			
18–29 yrs	91 (48)	47 (7)		48 (43–52)
30–39 yrs	40 (21)	45 (9)	- 4%	44 (39–48)
40–49 yrs	36 (19)	42 (7)	- 7%	40 (36–44)
50–61 yrs	23 (12)	38 (6)	- 10%	38 (34–41)
**Female:**	**113**			
18–29 yrs	40 (35)	34 (7)		38 (33–42)
30–39 yrs	37 (33)	36 (6)	+ 6%	34 (30–38)
40–49 yrs	16 (14)	31 (5)	- 14%	31 (26–35)
50–61 yrs	20 (18)	26 (4)	- 16%	29 (24–33)

SD, standard deviation; VO_2max_, maximal oxygen consumption during multistage 20-meter shuttle run test.

### Independent Predictors of VO_2max_

Results of the backward stepwise multiple linear regression analysis with VO_2max_ as dependent variable are presented in Table 5. Adjusted R^2^ of the model was high explaining 69% of variance in VO_2max_. In decreasing order, female gender, age, BMI, VPA in leisure-time, HR_max_-to-HR_rest_, occupational group 3 (vs. group 1), smoking, as well as sufficient MVPA on workdays contributed significantly to the model. In contrast, AEE / MPA / VPA / Steps during work-time, MPA / AEE / Steps in leisure-time, AEE / MPA / VPA / Steps and sufficient MVPA on non-working days, ex-smoking, occupational group 2 (vs. group 1) and HR_max_-to-HR_recovery_ were not found to be significant predictors of VO_2max_. Based on the results of the multiple linear regression analysis, this study has generated the following prediction equation:

$$\begin{array}{l} {\text{VO}}_{2\text{max}}\left[\text{ml}/\text{kg}/\text{min}\right]=\;63.006–\;\left(11.902\;\times \text{ gender};\text{ men }=\;1,\text{ women }=\;2\right)\;–\;\left(0.197\;\times \text{ age }\left[\text{yrs}\right]\right)\\ –\;\left(0.602\;\times \text{ BMI }\left[\text{kg}/{\text{m}}^{2}\right]\right)\;+\;\left(0.155\;\times \text{ VPA leisure}-\text{time }\left[\text{min}/\text{day}\right]\right)\;+\;\left(3.790\;\times {\text{ HR}}_{\text{max}}-\text{to}-{\text{HR}}_{\text{rest}}\right)\\ –\;\left(2.788\;\times \text{ occupational group};\text{ group }1\;=\;0,\text{ group }3\;=\;1\right)\;–\;(3.061\;\times \text{ smoking};\text{ never smokers }=\;0,\\ \text{current smokers }=\;1)\;+\;\left(2.506\;\times \text{ MVPA workday};\text{ insufficient }=\;1,\text{ sufficient }=\;2\right). \end{array}$$

**Table 5:** Backward stepwise multiple linear regression analysis with VO_2max_ as dependent variable.

Model: n = 273, Adjusted R^2^ = 0.69	B	SE B	β	p-value
*Constant*	*63*.*006*	*4*.*882*		*<0*.*001*
Gender (male vs. female)	-11.902	0.811	-0.622	<0.001
Age [yrs]	-0.197	0.029	-0.264	<0.001
BMI [kg/m^2^]	-0.602	0.112	-0.220	<0.001
VPA leisure-time [min/day]	0.155	0.027	0.212	<0.001
HR_max_-to-HR_rest_	3.790	0.720	0.192	<0.001
Occupational group (group 1 vs. group 3)	-2.788	0.842	-0.141	<0.001
Smoking (never vs. current smokers)	-3.061	0.811	-0.133	<0.001
MVPA workday (insufficient vs. sufficient)	2.506	1.001	0.100	0.013

B, unstandardized regression coefficient; β, standardized beta coefficient; BMI, body mass index; HR_max_-to-HR_rest_, ratio of maximum to resting heart rate; MVPA workday (insufficient vs. sufficient), comparison of insufficient (<30 min per day) to sufficient (≥30 min per day) moderate-to-vigorous physical activity calculated from bouts of ≥10 min on workdays; Occupational group (group 1 vs. group 3), comparison of group 1 with low occupational physical activity to group 3 with high occupational physical activity; SE, standard error; VO_2max_, maximal oxygen consumption during multistage 20-meter shuttle run test; VPA, physical activity duration at vigorous (≥6 METs) intensity. Excluded variables were: AEE / MPA / VPA / Steps during work-time, AEE / MPA / Steps in leisure-time, AEE / MPA / VPA / Steps on non-working days, ex-smokers (vs. never smokers), occupational group 2 (vs. group 1), HR_max_-to-HR_recovery_, MVPA non-working days (insufficient vs. sufficient).

## Discussion

### Main Findings

This cross-sectional study found that men had higher levels of both, physical activity and VO_2max_ than women. In general, physical activity was more common during workdays than during non-working days, especially among men. In addition, on workdays, mean VO_2max_ was significantly higher in participants, who fulfilled the global recommendations on physical activity compared to insufficiently active counterparts. Multiple linear regression analysis with VO_2max_ as dependent variable showed significant positive associations of VO_2max_ with LTPA at high intensities and with sufficient MVPA on workdays, but not with OPA at any intensity. Female gender, age, BMI, the ratio of maximum to resting heart rate, occupational group 3 (vs. group 1), and smoking appeared as additional independent predictors of VO_2max_.

### Predictors of VO_2max_

The present results are in line with existing knowledge that females usually present lower levels of physical activity and VO_2max_ than males. The regression model with objective SenseWear activity data has shown that female gender is the strongest predictor of VO_2max_. The gender difference in VO_2max_ can partly be explained by a higher percentage of body fat and a lower hemoglobin concentration in females compared to males. Furthermore, men have a higher proportion of muscle mass and thus more mitochondria than females [8]. The decline of VO_2max_ with age in the present study is comparable to previous studies of Jackson *et al*. [6, 7]. In this study, participants younger than 50 years had a mean decline <10% per decade and those older than 50 years a decline >10% per decade. As shown by previous studies, measured VO_2max_ presented a significant negative association with BMI. The ratio of HR_max_-to-HR_rest_ contributed positively to the model, which is in line with a study of well-trained men by Uth *et al*. [26], who observed highly significant correlations between measured VO_2max_ and the Heart Rate Ratio Method. Consistent with previous investigations, measured VO_2max_ was negatively associated with smoking [32]. In this study, only VPA in leisure-time contributed significantly to the regression model and showed a positive association with VO_2max_. Kenney *et al*. [8] stated that the higher the initial state of fitness, the smaller is the relative improvement for the same volume of training. Subjects with a mean baseline VO_2max_ of 40–51 ml/kg/min required an intensity of at least 45% of oxygen uptake reserve (≙ 4.7–6.1 METs) to improve VO_2max_ [33]. Since VO_2max_ values of a large proportion of the present sample were within this range (mean VO_2max_ = 40 ml/kg/min, SD 10), this study could confirm the statement, even if the threshold of 4.7–6.1 METs still falls within the range of MPA, but at the upper limit. Contrary to expectation, OPA at any intensity was not positively associated with VO_2max_. A possible reason could be that OPA despite its long duration is more intermittent and not as effective as LTPA, which in general is planned, structured, often short, of high intensity and very efficient [29]. However, the present results are contradictory to previous findings. Hammermeister *et al*. [34] and Jang *et al*. [12] suggested that OPA could have a significant effect on VO_2max_. Hirai *et al*. [35] also found a significant relationship between work form and VO_2max_ in male workers. The reason that they found positive associations between OPA and VO_2max_ might be that they used simple self-reported questionnaires for assessing OPA. Miller & Brown [36] stated that measuring work-related activity is problematic, because the intermittent and unstructured nature of most work-related activities makes self-report difficult. The finding that subjects in occupational group 3 with high OPA had a lower VO_2max_ than those in group 1 with low OPA could be explained by the lack of motivation to engage in high-intensity sports and exercises after a tiring work-shift, since only VPA in leisure-time was found to be predictive for VO_2max_.

### Generalizability of Results

The present study included a wide range of manual and non-manual employees and represented a typical cross-section of the Swiss working population. Mean BMI, percentage of overweight, obesity, as well as gender distribution were comparable to the Swiss working population in 2014 [37]. Furthermore, VO_2max_ values in this study were in high agreement with a previous population-based study in US employees [38]. This applies to mean total VO_2max_ as well as mean VO_2max_ in men and women. Physical activity data measured by the SenseWear Mini Armband were comparable to a recent study that objectively measured physical activity in 9554 Finnish employees with the Firstbeat Bodyguard device (dedicated device for beat-by-beat 24 h heart rate and heart rate variability measurement) [39]. In both studies, most physical activity was performed within the moderate-intensity range, and activity levels at all intensities were higher in males compared to females. Contradictory to this study was that on non-working days men accumulated more physical activity at all intensities than on workdays. Concerning women, the present results were consistent with their findings. In both studies, women showed more MPA on non-working days compared to workdays, while it was the contrary for HPA and VHPA.

### Findings in Relation to Physical Activity Recommendations

In this study, 84% and 75% of participants fulfilled the activity recommendations of ≥30 min MVPA per day on workdays and non-working days, respectively. These findings were slightly higher compared to a survey in the Swiss population from 2012, where 72% were sufficiently active in their leisure-time [40]. However, in the survey a self-reported questionnaire was used to assess physical activity and only LTPA was considered. This might explain the higher percentage of participants fulfilling the activity recommendations in the present study compared to the Swiss survey of 2012. An explanation for the findings that LTPA on workdays was predictive for VO_2max_ and participants being sufficiently active on workdays presented a higher VO_2max_, could be that on workdays employees only have a little time window for exercising outside work, which increases the density of activities. In contrast, on non-working days physical activity may be more unstructured and less efficient because of the extended time availability (lower density of physical activity).

### Clinical Implications

The potential health benefits of good cardiorespiratory fitness (high VO_2max_ value) are well known. It reduces the risk of various diseases in the general population. This investigation has shown that OPA does not contribute to an improvement of VO_2max_. In contrast, to maintain or improve VO_2max_, intensive physical exercise in leisure-time in the range of high-to-very high intensity is required, as for example athletic cycling, soccer, martial arts, squash, inline skating or aerobics [41]. A low VO_2max_ and insufficient physical activity have a strong impact on individuals’ wellbeing and all-cause mortality [2, 3]. Based on the present findings, it may be recommended to implement an attractive and intensive sports program at the workplace, such as lunch-time or after-work exercise in order to improve the overall health in the working population.

### Strengths and Limitations of the Study

The present study had several strengths. The study sample included a wide range of manual and non-manual employees and represented a typical cross-section of the Swiss working population. Furthermore, the measurement of physical activity and VO_2max_ was conducted with objective instruments. The SenseWear Mini Armband promises an accurate assessment of physical activity under non-ambulatory conditions. The inclusion of thermal- and perspiration-related sensors in addition to the three-axis accelerometer provides a way to detect subtle increases in physical activity associated with low-intensity activities [20]. Furthermore, the detection of non-wearing, resting and sleep time allow for more confidence in data consistency. However, there are also some limitations associated with the Sense Wear Mini Armband. The device has been shown to underestimate physical activity at very high intensities (>10 METs) [42]. This may explain why in the present study only few subjects had activities above this intensity threshold (VHPA). Though, the relationship between VO_2max_ and vigorous LTPA is expected to be even stronger with reliable measurements of VHPA. Moreover, the Sense Wear Mini Armband was found to underestimate activities involving purely lower extremities, such as cycling, because of its wearing position on the upper arm [43]. In addition, it is not waterproof and lacks to detect water-based activities. A strength of this study were the strict exclusion criteria for recording days (e. g. measurement periods of <22 h per day or <12 h during wake time were excluded from analysis). Thus, the recordings covered well typical workdays and non-working days. Nevertheless, the present study also had some weaknesses. Since only 4% of women participated in group 3 (high OPA), there is a need for further investigations to focus on females in this subgroup. Another limitation was that VO_2max_ values were not directly measured, but predicted from the score, which participants reached in the 20-meter shuttle run test. For direct measurement of VO_2max_, spiroergometry is considered to be the gold standard [44]. However, spiroergometry is labour-intensive, requires trained staff and is therefore not feasible for assessing VO_2max_ in large populations [45]. A recent study confirmed the validity of the 20-meter shuttle run test and concluded that it can accurately predict VO_2max_ in healthy adults [46]. The results revealed significant correlations between the number of shuttles in the 20-meter shuttle run test and directly measured VO_2max_ (r = 0.87, p<0.05) as well as the velocity at which VO_2max_ occurred (r = 0.93, p<0.05) [46]. The present study was cross-sectional in nature, which provided a snapshot of the relation between physical activity and VO_2max_. However, cause-effect-relationships are difficult to ascertain. To overcome this limitation in future, longitudinal intervention studies are required.

## Conclusions

The key finding of this cross-sectional investigation is that VO_2max_ was not positively associated with OPA in a representative cohort of healthy Swiss employees, when objectively measured with the SenseWear Mini Armband. In accordance with existing knowledge, VO_2max_ showed a positive association with LTPA at vigorous intensity and with sufficient MVPA on workdays. Furthermore, female gender, age, BMI, the ratio of maximum to resting heart rate, occupational group 3 (vs. group 1), and smoking were identified as independent predictors of VO_2max_. These findings provide the basis and therefore will be of great value for developing evidence-based strategies to improve cardiorespiratory fitness in the working population.

## Supporting Information

S1 Table Professions of study subjects, stratified according to occupational group and category. (DOCX)

S1 Dataset Excel file of personal an job-related factors. (XLSX)

S2 Dataset Excel file of body measurements and 20-meter shuttle run. (XLSX)

S3 Dataset Excel file of SenseWear Mini Armband activity parameters. (XLSX)

S1 Protocol Study protocol approved by the local ethics committee. (DOC)
