# Supplementary material for: Association of Occupational and Leisure-Time Physical Activity with Aerobic Capacity in a Working Population
Source: PLoS One. 2017 Jan 3;12(1):e0168683. doi: 10.1371/journal.pone.0168683 (PMC5207528; doi:10.1371/journal.pone.0168683)
Supplement: S1 Table — (DOCX) [file pone.0168683.s001.docx]

| **S1 Table. Professions of study subjects, stratified according to occupational group and category. Group with low OPA.** | | | |
| --- | --- | --- | --- |
| Group | Occupational category | Profession | N |
| Low OPA  (N = 101) | Managers (N = 25) | Business economy manager | 2 |
|  |  | City gardener manager | 1 |
|  |  | Clerc manager | 1 |
|  |  | Departement head | 2 |
|  |  | Foreman | 4 |
|  |  | Human ressources manager | 1 |
|  |  | Iinformation scientist manager | 1 |
|  |  | Logistic manager | 1 |
|  |  | Machine design manager | 1 |
|  |  | Merchant manager | 4 |
|  |  | Nurse manager | 1 |
|  |  | Nutrition manager | 1 |
|  |  | Operating muesum manager | 1 |
|  |  | Patient service manager | 1 |
|  |  | Project manager | 1 |
|  |  | Quality management manager | 2 |
|  | Scientists (N = 35) | Business economist | 2 |
|  |  | Business economist consultant | 1 |
|  |  | Certified accountant | 1 |
|  |  | Doctor | 7 |
|  |  | Economist | 1 |
|  |  | Economy lawyer | 1 |
|  |  | Engineer | 3 |
|  |  | Human resource consultant | 2 |
|  |  | Information scientist | 3 |
|  |  | Intensive care expert | 1 |
|  |  | Interior decorator | 1 |
|  |  | Marketing employee | 1 |
|  |  | Medical assistant | 4 |
|  |  | Movement scientist | 1 |
|  |  | Operating manager | 1 |
|  |  | Psychologist | 1 |
|  |  | Scientific assistant | 3 |
|  |  | Scientist | 2 |
|  | Office workers (N = 41) | Accounting clerc | 4 |
|  |  | Bank employee | 1 |
|  |  | Clerc | 15 |
|  |  | Insurance employee | 1 |
|  |  | Marketing | 1 |
|  |  | Merchant | 19 |

| **S1 Table. Professions of study subjects, stratified according to occupational group and category. Group with moderate OPA.** | | | |
| --- | --- | --- | --- |
| Group | Occupational category | Profession | N |
| Moderate OPA  (N = 102) | Technicians (N = 76) | Assembly operator | 1 |
|  |  | Biomedical analyst | 3 |
|  |  | Cook | 2 |
|  |  | Design engineer | 1 |
|  |  | Electrician | 1 |
|  |  | Elevator service technician | 1 |
|  |  | Equipment engineer | 1 |
|  |  | Ergo therapist | 4 |
|  |  | Graduate nurse | 14 |
|  |  | Medical office assistant | 1 |
|  |  | Midwife | 4 |
|  |  | Nurse | 2 |
|  |  | Nutritionist | 2 |
|  |  | Pharma assistant | 1 |
|  |  | Physiotherapist | 15 |
|  |  | Polygraph | 1 |
|  |  | Safety engineer | 1 |
|  |  | Salesclerc | 5 |
|  |  | Sanitary installer | 0 |
|  |  | Set-up man | 1 |
|  |  | Social education worker | 1 |
|  |  | Sports teacher | 6 |
|  |  | Store detective | 1 |
|  |  | Teacher | 3 |
|  |  | Technical employee | 3 |
|  |  | Technical interior designer | 1 |
|  |  | Technical sterilization assistant | 1 |
|  |  | Trustee | 1 |
|  | Service workers (N = 20) | Coiffeur | 18 |
|  |  | Police officer | 2 |
|  | Machine operators (N = 4) | Crane operator | 2 |
|  |  | Truck driver | 2 |

| **S1 Table. Professions of study subjects, stratified according to occupational group and category. Group with high OPA.** | | | |
| --- | --- | --- | --- |
| Group | Occupational category | Profession | N |
| High OPA  (N = 100) | Agricultural workers (N = 9) | Arborist | 2 |
|  |  | Forest worker | 6 |
|  |  | Horticulturist | 1 |
|  | Craftsmen (N = 82) | Bicycle mechanic | 1 |
|  |  | Bricklayer | 10 |
|  |  | Building worker | 3 |
|  |  | Building worker foreman | 3 |
|  |  | Caretaker | 4 |
|  |  | Caretaker | 1 |
|  |  | Carpenter | 8 |
|  |  | Ccarpenter | 3 |
|  |  | Coachbuilder | 3 |
|  |  | Electro installer | 1 |
|  |  | Logistician | 21 |
|  |  | Mechanic | 10 |
|  |  | Painter | 1 |
|  |  | Plumber | 3 |
|  |  | Road builder | 2 |
|  |  | Sanitary installer | 3 |
|  |  | Telematics | 1 |
|  |  | Warehousekeeper | 4 |
|  | Labourers (N = 9) | Assembly operator assistant | 6 |
|  |  | Bricklayer assistant | 1 |
|  |  | Building worker assistant | 1 |
|  |  | Machine operator assistant | 1 |
